# Intestinal inflammation and increased intestinal permeability in *Plasmodium chabaudi* AS infected mice

**DOI:** 10.12688/wellcomeopenres.17781.1

**Published:** 2022-04-13

**Authors:** Jason P Mooney, Sophia M DonVito, Rivka Lim, Marianne Keith, Lia Pickles, Eleanor A Maguire, Tara Wagner-Gamble, Thomas Oldfield, Ana Bermejo Pariente, Ajoke M Ehimiyien, Adrian A Philbey, Christian Bottomley, Eleanor M Riley, Joanne Thompson

**Affiliations:** 1 Institute of Immunology and Infection Research, University of Ediburgh, Edinburgh, Midlothian, EH93JT, UK; 2 Division of Infection and Immunity, The Roslin Institute, University of Edinburgh, Edinburgh, EH25 9RG, UK; 3 Editorial Team, F1000 Ltd., London, UK; 4 Department of Veterinary Medicine, Ahmadu Bello University, Zaria, Nigeria; 5 Easter Bush Pathology, Royal (Dick) School of Veterinary Studies, University of Edinburgh, Edinburgh, United Kingdom., Edinburgh, EH25 9RG, UK; 6 Department of Infectious Disease Epidemiology, London School of Hygiene and Tropical Medicine, London, WC1E 7HT, UK

**Keywords:** malaria, plasmodium, intestine, permeability, enteritis

## Abstract

**Background: **Gastrointestinal symptoms are commonly associated with acute *Plasmodium* *spp* infection. Malaria-associated enteritis may provide an opportunity for enteric pathogens to breach the intestinal mucosa, resulting in life-threatening systemic infections.

**Methods: **To investigate whether intestinal pathology also occurs during infection with a murine model of mild and resolving malaria, C57BL/6J mice were inoculated with recently mosquito-transmitted *Plasmodium chabaudi* AS. At schizogony, intestinal tissues were collected for quantification and localisation of immune mediators and malaria parasites, by PCR and immunohistochemistry. Inflammatory proteins were measured in plasma and faeces and intestinal permeability was assessed by FITC-dextran translocation after oral administration.

**Results: **Parasitaemia peaked at approx. 1.5% at day 9 and resolved by day 14, with mice experiencing significant and transient anaemia but no weight loss. Plasma IFN-γ, TNF-α and IL10 were significantly elevated during peak infection and quantitative RT-PCR of the intestine revealed a significant increase in transcripts for *ifng* and *cxcl10*. Histological analysis revealed parasites within blood vessels of both the submucosa and intestinal villi and evidence of mild crypt hyperplasia. In faeces, concentrations of the inflammatory marker lactoferrin were significantly raised on days 9 and 11 and FITC-dextran was detected in plasma on days 7 to 14. At day 11, plasma FITC-dextran concentration was significantly positively correlated with peripheral parasitemia and faecal lactoferrin concentration.

**Conclusions: **In summary, using a relevant, attenuated model of malaria, we have found that acute infection is associated with intestinal inflammation and increased intestinal permeability. This model can now be used to explore the mechanisms of parasite-induced intestinal inflammation and to assess the impact of increased intestinal permeability on translocation of enteropathogens.

## Introduction

Symptomatic malaria parasite infection is characterised by a cyclical fever, anaemia, and malaise with *Plasmodium spp*-infected red blood cells (RBCs) detectable in the peripheral circulation. Gastrointestinal symptoms are commonly noted in malaria patients, with diarrhoea reported in both travelers and those residing in malaria-endemic areas (systematically reviewed, ( Sey *et al.*, 2020)). For example, diarrhoea was reported in 25% of 451 Ugandan children hospitalised with malaria, significantly more frequently than among malaria-uninfected hospitalised children (11%) ( Lo Vecchio *et al.*, 2021) and treatment with antimalarial drugs can resolve diarrhoeal symptoms within 48 hours ( Lo Vecchio *et al.*, 2021; Sowunmi *et al.*, 2000), suggesting, but not proving, a causal association.

The primary role of the intestine is digestive, absorbing both water and nutrients whilst creating a barrier to invasion by microorganisms including pathogens. Disturbance of normal intestinal function can result in diarrhoea; a diverse clinical presentation being either watery (e.g. osmotic or secretory) or exudative (i.e. with mucus, blood and cellular discharge). Gastrointestinal pathogens, (protozoal, bacterial and viral) are highly prevalent in malaria-endemic areas and episodes of diarrhoea are common, especially among children ( Troeger *et al.*, 2018). It can, therefore, be challenging to determine whether an episode of diarrhoea is caused by a concurrent malaria infection or is simply coincidental.

One common intestinal pathogen, non-Typhoidal Salmonella (NTS), a particularly frequent cause of invasive bacterial disease (invasive NTS, iNTS) in sub-Saharan Africa resulting in considerable morbidity and mortality (reviewed ( Takem *et al.*, 2014) is, however, significantly more common in people with, or recently recovered from, a clinical episode of malaria than among those with no recent history of malaria infection ( Biggs *et al.*, 2014; Park *et al.*, 2016; Scott *et al.*, 2011). Whilst the features of diarrhoea associated with *Plasmodium* infection remain ill-defined ( Sey *et al.*, 2020), an association with increased intestinal permeability ( Pongponratn *et al.*, 1991) and decreased absorption of vitamin B _12_ and D-xylose ( Karney & Tong, 1972) has been observed. Furthermore, autopsies of individuals dying from malaria have revealed intestinal haemorrhages ( Dudgeon & Clarke, 1919) and some evidence of sequestered infected RBCs in villous capillaries ( Seydel *et al.*, 2006; Pongponratn *et al.*, 1991). Given the continuing world-wide burden of clinical malaria ( Organization, 2021), the additional burden of subclinical malaria infections ( Stresman *et al.*, 2020), and the burden of enteropathogenic infections ( Geus *et al.*, 2019), it is important to understand associations between these infections at the intestinal level.

To date, our understanding of malaria-associated intestinal disturbance comes largely from virulent murine models of severe and non-resolving *Plasmodium* infection. For example, *Plasmodium yoelii* infection has been associated with mild caecal inflammation, dysbiosis of the intestinal flora, increased colonisation with *E. coli* and NTS, and increased intestinal permeability (as measured by lactose:mannitol absorption ratios) ( Chau *et al.*, 2013; Mooney *et al.*, 2015). Small intestinal dysbiosis and pathology has also been observed in *Plasmodium berghei* ANKA infected mice ( Shimada *et al.*, 2019; Taniguchi *et al.*, 2015), and traditional serial-blood passaged *Plasmodium chabaudi* infection has been associated with increased cellular influx in the jejunum and increased intestinal permeability ( Alamer *et al.*, 2019). Taking these findings together, a picture is emerging in which severe, acute *Plasmodium spp.* infection in mice induces intestinal inflammation leading to dysbiosis, increased intestinal permeability and increased colonisation by intestinal pathogens. However, the molecular and cellular processes underlying these intestinal responses, particularly whether they are driven by systemic or local inflammation, are unknown.

These murine models of malaria are characterized by high parasitaemia and moderate to severe symptoms, however, and are therefore less representative of human malaria parasite infections in endemic settings. Therefore, to evaluate the intestinal response in a more physiologically-relevant model of mild to moderate malaria during acute and resolving infection, we have used the recently-transmitted model, in which mosquito transmission attenuates parasite virulence and modifies the host immune response ( Spence *et al.*, 2013; Spence *et al.*, 2015). Moreover, we used a fluorescently-tagged *P. chabaudi* AS line to facilitate imaging of infected RBCs to resolve whether intestinal inflammation is directly associated with parasite sequestration.

## Methods

### Ethical Statement and mouse procedures at the University of Edinburgh

This study was reviewed and approved by the Ethical Review Body of the University of Edinburgh. All procedures were carried out in accordance with the UK Home Office regulations (Animals Scientific Procedures Act, 1986) under Project Licence number P04ABDCAA. Throughout this study, all efforts were made to reduce animal usage and ameliorate harm to animals. Mice were housed in the University of Edinburgh Licenced Animal Facilities 60/2605), and all animal procedures were performed in laboratories within the animal facilities. Six to eight weeks old female C57BL/6J mice were purchased from Charles River (Tranent, UK). All animals were maintained with at least one companion, randomly housed in individually ventilated cages furnished with autoclaved woodchip, fun tunnel and tissue paper at 21 ± 2°C, under a reverse light-dark cycle (light, 19.00 – 07.00; dark, 07.00 – 19.00) at a relative humidity of 55 ± 10% in a specified pathogen free facility. Mice were housed under these light-dark cycle conditions to allow collection of *P. chabaudi* trophozoites prior to schizogony at 13.00–15.00 hrs, and were allowed to adapt to a reverse-light schedule for at least 7 days before *P. chabaudi* infection. They were fed *ad libitum* an autoclaved dry rodent diet (RM3, Special Diets Services, UK), along with autoclaved water. Animals were monitored according to institutional guidelines, with routine daily health checks and increased monitoring during *P. chabaudi* infection. Euthanasia was performed by cervical dislocation at the end of phenotypic experiments, or by exsanguination under anaesthesia (pentobarbital sodium; Euthatal). This specific method of anaesthesia reduces animal suffering whilst maximising blood volume obtained.

### Infection and monitoring

The C57Bl/6 *mus musculus*- *Plasmodium chabaudi chabaudi* AS animal model of malaria was chosen to minimize host genetic variability and to obtain robust infections with a very low incidence of severe disease. Animals were inoculated i.p. with 1×10 ^5^ PcAS-GFP or PcAS-mCherry-infected RBCs (iRBC) that had been blood passaged 3–6 times since primary infection by mosquito; deemed ‘recently mosquito-transmitted PcAS infection’, as previously described ( Spence *et al.*, 2013). GFP or mCherry are constitutively expressed in the cytoplasm of these parasites at all stages of development ( Marr *et al.*, 2020).

In total, 208 mice were used in this study, in 8 experiments with groups of 4–7 mice, to provide statistical significance. Mice were infected with GFP-expressing PcAS (4 experiments, n=79), mCherry-expressing PcAS (4 experiments, n=51), or were uninfected controls (n=58). For each experiment, 2 mice were used to expand frozen stocks of stabilate parasites, (16 mice total). Four mice were excluded; three inoculated mice which were uninfected and one with an unexpectedly high parasitemia. For each experimental readout per time-point, two independent experiments were performed.

Mice were weighed and monitored for haemoglobin concentration and parasitaemia by tail snip blood sampling at 18–21hrs of the blood-stage life-cycle for optimal detection of circulating trophozoites, as described previously ( Marr *et al.*, 2020). Parasitemia was determined by flow cytometric analysis; diluting 1µL of tail blood in 1mL of Dulbecco's phosphate-buffered saline (dPBS, Gibco, UK) containing 5 IU mL ^-1^ heparin sodium (L6510, VWR), and then diluting a further 1:5 prior to acquisition on a BD Fortessa (Becton Dickinson, UK). At least 100,000 events were analysed per sample; gates were set using uninfected control blood using FlowJo V10 (Tree Star), as previously shown ( Marr *et al.*, 2020). Processing of control blood was performed prior to that from infected mice to minimise potential cross-contamination upon data acquisition. Haemoglobin concentration (Hb, g/L) was measured using a Hemocue Hb201+ (Radiometer, Sweden). Weight change was calculated as a proportion of an individual’s pre-infection weight, with measurements taken prior to tail snips. At various days post infection, mice were euthanized (at the time of predicted schizogony) by exsanguination under anaesthesia (pentobarbital sodium; Euthatal) for tissue and/or blood collection following cardiac puncture. Data are shown as ‘day post infection’. As blood sampling was timed to coincide with the presence of circulating trophozoites (i.e. before schizogony, which marks the completion of a replicative cycle) the number of completed replicative cycles is one less than the number of days pi.

### Detection of plasma proteins

Cardiac blood was collected into 5µL of heparin sodium (5 IU ml ^-1^), centrifuged at 10,000g for 5 min, and plasma stored at -70°C for subsequent analysis. For multiplex analysis, a magnetic Luminex assay (LXSAMSM-7, R&D systems, UK) was performed according to the manufacturer’s instructions for IFN-γ (BR33), TNF-α (BR14), and IL-10 (BR28), using undiluted samples and analysed on a Bio-Plex 200 (Bio-Rad, USA). For IFN-γ analysis by enzyme-linked immunosorbent assay (ELISA), plasma was diluted 1:2 and assayed with the mouse IFN-γ ELISA MAX deluxe (430804, BioLegend, UK) according to the manufacturer’s instructions. Samples which gave values below the detectable range were reported at the limit of detection for each analyte.

### qPCR analysis of mouse intestines

At necropsy, the intestines were divided into five equal lengths (three for the small intestine, two for the large), and cleaned of contents by flushing with dPBS. Tissue was then immersed in 1mL RNAlater (Sigma-Aldrich, UK) and stored at -70°C after chilling according to the manufacturer‘s instructions. For isolation of RNA, tissue was transferred to 2mL FastPrep Lysing Matrix D tubes (MP Biomedicals) containing 1mL of TRIzol (Invitrogen). Tissues were then homogenized using a Precellys 24 tissue homogenizer (Bertin instruments) at 30sec on high speed, followed by phenol/chloroform extraction with TRIzol according to the manufacturer’s instructions. Residual DNA was then removed (AM1906, Ambion/Thermo-fisher). Purified RNA was measured using a NanoDrop spectrophotometer and diluted to 100 ng/mL prior to cDNA synthesis using the AffinityScript Multiple Temperature cDNA synthesis kit (200436, Agilent), according to the ‘1 ^st^ strand cDNA synthesis’ manufacturer’s protocol using 1µg RNA in a 40μL volume. For each sample, 2µL of the cDNA was transferred to a 96-well plate with 18µL of mastermix (dispensed by robot, Corbett CAS-1200) containing 10µL Brilliant III Sybrgreen Ultrafast Mastermix (600882, Agilent), 6.4µL ultrapure water (Gibco), and 0.8µL of both forward and reverse primers (diluted to 10µM) for each gene target (sequences listed in Table 1). Samples were run on a CFX96 Real-Time PCR Detection System (Bio-Rad, USA) at 96°C for 3min, followed by 40 cycles of 96°C for 5 sec and 60°C for 10 sec, and data acquisition. Data were analyzed using the comparative threshold cycle (C _T_) method. Target gene transcription of each sample was normalized to the respective levels of β-actin mRNA and represented as fold change over gene expression in control animals, as described previously ( Lokken *et al.*, 2014). To summarise, to calculate the relative fold gene expression, an individual reference gene Ct value (β-Actin) is subtracted from the target gene Ct value (ΔCt), with the mean of control samples then subtracted (ΔΔCt), and finally the value is taken to two to the negative power (2- ^ΔΔCt^).

**Table 1.** qPCR primers used in the study.

Species	Target	Forward (sense)	Reverse (antisense)
Mus Musculus	*Ifng*	CAACAGCAAGGCGAAAAAGGATGC	CCCCGAATCAGCAGCGACTCC
Mus Musculus	*Cxcl10*	GGACTCAAGGGATCCCTCTCG	GGCTCGCAGGGATGATTTCAA
Mus Musculus	*Cd68*	TGTCTGATCTTGCTAGGACCG	GAGAGTAACGGCCTTTTTGTGA
Mus Musculus	*Il10*	GGTTGCCAAGCCTTATCGGA	ACCTGCTCCACTGCCTTGCT
Mus Musculus	*Tnfa*	GCAGGTTCTGTCCCTTTCACTCACTG	TGGAAGCCCCCCATCTTTTGG
Mus Musculus	*Lcn2*	ACATTTGTTCCAAGCTCCAGGGC	CATGGCGAACTGGTTGTAGTCCG
Mus Musculus	*Cxcl1*	GCTTGCCTTGACCCTGAAGCTC	TGTTGTCAGAAGCCAGCGTTCAC
Mus Musculus	*Mip2*	CGCCCAGACAGAAGTCATAGCCAC	TCCTTTCCAGGTCAGTTAGCCTTGC
Mus Musculus	*Il27*	TTCCCAATGTTTCCCTGACTTT	AAGTGTGGTAGCGAGGAAGCA
Mus Musculus	*bActin*	AGAGGGAAATCGTGCGTGAC	CAATAGTGATGACCTGGCCGT
*Plasmodium chabaudi*	*18S rDNA*	AAGCATTAAATAAAGCGAATACATCCTTAT	GGGAGTTTGGTTTTGACGTTTATGCG

### Histology and Immunohistochemistry

Rolls of cleaned intestinal tissue were fixed immediately in 10% PBS-buffered formalin, followed by embedding into paraffin prior to sectioning into 4–5µm slices onto charged slides. Slides were deparaffinised and rehydrated using an AutoStainer XL (Leica) prior to staining. For visualisation of *Plasmodium* parasites, immunohistochemistry was performed targeting GFP in MT-PcAS-GFP-infected mice. Antigen retrieval was achieved by autoclaving (121 ^o^C, 45min) in TRS (pH 6.1; S169984-2, Agilent Dako), followed by washing in PBS/0.1% Tween20. Slides were blocked with 3% hydrogen peroxide for 10min, followed by non-specific horse serum matched to the secondary antibody for 15min, followed by blocking with avidin and biotin for 15min (927301, Bio Legend). Slides were incubated with goat anti-GFP (AF4240, R&D Systems) diluted 1:500 in PBS/0.1%Tween+1%FBS) in a humified chamber at 4 ^o^C overnight. After 3 x 15min washes in PBS/0.1%Tween, slides were incubated with biotinylated horse anti-goat IgG H+L (BA-9500, Vector Laboratories) diluted 1:500 for 1h at room temperature. Normal goat IgG (AB-108-AC, R&D Systems) or no primary antibody were used as controls. Finally, slides were stained with DAB (SK-4100, Vector Laboratories) using an ABC reagent kit (32020, Thermo Fisher), according to the manufacturers’ instructions with substrate development for 10min, and counterstained with hematoxylin (3136, Sigma Aldrich) using an autostainer XL (Leica).

Villous height and crypt depth were measured on haematoxylin and eosin-stained sections scanned at 40x with a NanoZoomer (Hamamatsu Photonics, Japan) and analysed with QuPath Software (v0.2.3) ( Bankhead *et al.*, 2017), an open platform for bioimage analysis. Quality assessment scoring (i.e. focus, small artefacts, orientation of the villi) was performed on randomized and blinded scans, followed by collection of 30 measurements of pairs of neighboring crypts and villi using the line tool. Each intestinal roll was divided into three sections (proximal, medial, and distal), with 10 crypts and villi measured in each area. Villus height to crypt depth ratio (Vh:Cd) for each neighbouring pair was calculated, then averaged for either the entire small intestine or each section.

### Assessment of intestinal permeability

Intestinal permeability was assessed as described previously ( Alamer *et al.*, 2019; Denny *et al.*, 2019; Taniguchi *et al.*, 2015), with modifications. Food was withdrawn from cages for 5 hours prior to oral gavage with 0.1mL of 4-kDA fluorescein isothiocyanate (FITC) dextran (FD4-1g, Sigma) diluted to 25mg/mL in water, with the time of gavage recorded for each animal. Food was returned after gavage and mice were culled exactly 1 hour post gavage ( Volynets *et al.*, 2016; Woting & Blaut, 2018). 100µL of plasma (collected as described above) was placed in a black, flat-bottomed 96-well plate, and fluorescence intensity at 520nm measured after excitation at 485nm in a FLUOstar Omega microplate reader (BMG Labtech). FITC-dextran concentrations were calculated from a standard curve of 10-fold serial dilutions of FITC-dextran standard and analysed using Microsoft Excel.

### Faecal inflammatory proteins

Large intestines were excised from anus to caecum, split open with scissors and the contents collected with a blunt metal edge into a 2mL eppendorf tube. Samples were placed at -70°C until processing. Contents were weighed and 0.5mL of ‘faecal buffer’ (0.5% anti-protease cocktail (P8340, Sigma) in dPBS) added. Samples were allowed to rest for 30min at 4 ^o^C, then placed on a vortex adapter for 30min with continuous shaking, as described previously ( Fidler *et al.*, 2020). Faecal homogenates were centrifuged at 8,000g for 5min and 250μL of supernatant was stored at -70°C. Mouse proteins in faecal supernatants were enumerated by ELISA for IgA (88-50450, ThermoFisher), calprotectin (E1484Mo, Bioassay Technology Laboratory) and lactoferrin (EM1196, FineTest), according to the manufacturer’s instructions. Faecal supernatants were diluted 1:2 for lactoferrin detection and 1:400 for IgA, and were undiluted for calprotectin measurements. To measure residual FITC-dextran fluorescence, faecal supernatants were centrifuged a second time at 2,000g for 5min and then diluted 1:4 in water prior to reading at 485/520nm, as outlined above.

### Data analysis

Markers of inflammation were compared between mice culled at 4, 7, 11 and 14 days post infection and uninfected control mice. Each post-infection group was compared with the control group using Dunnett’s test to account for multiple testing. Where necessary the data were log-transformed to improve symmetry and when there was evidence of heterogeneity in variance between the groups, Dunn’s test with Bonferroni adjustment for multiple testing was used instead of Dunnett’s test. Correlations between membrane permeability (FITC-Dextran concentration) and parasite load or fecal lactoferrin were assessed using Pearson’s correlation coefficient. All statistical analyses were performed, and graphs made, using GraphPad Prism (v 8.2.1 or v 9.1.0). A p value of <0.05 was considered statistically significant.

## Results

**Figure 1.**
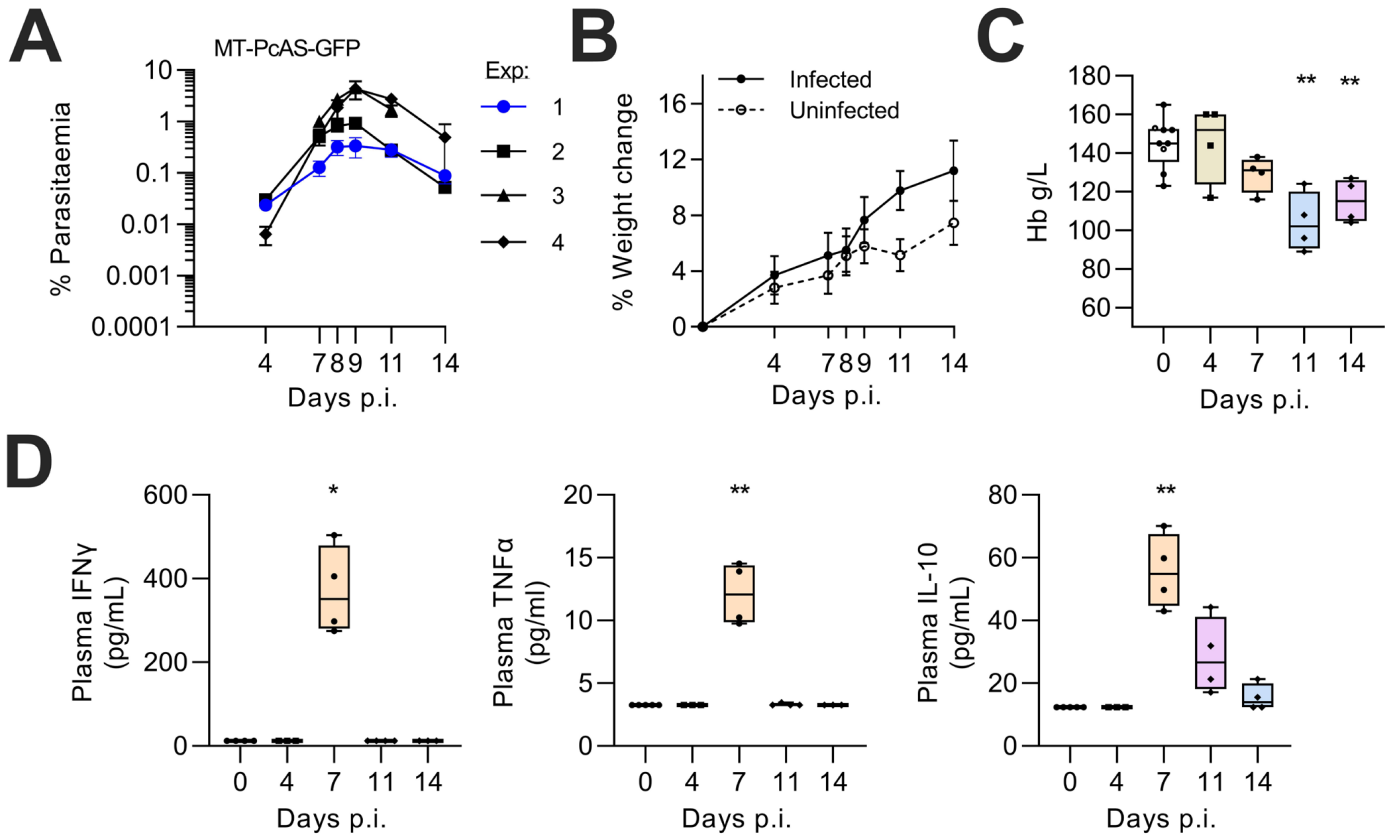
Anaemia and systemic inflammation during *P. chabaudi* infection in mice. Female C57BL/6 mice were infected with 1x10 ^5^ rMT-PcAS-GFP iRBC. ( **A**) Peripheral parasitaemia in tail blood was determined by flow cytometry. Data from four replicate experiments, n=4–5 mice per experiment, with Experiment 1 highlighted (blue line). Data shown as a mean±SEM. All data in panels B-D are drawn from experiment 1. ( **B**) Percentage increase in weight (mean±SEM) over time for infected (n=4) or uninfected (n=6) mice. ( **C**) Haemoglobin (g/L) from tail blood was determined by haemocue fluorometer from one representative experiment (n=4 per time-point, with 5 controls) with data shown as a box-whisker plot with dots representing individual mice. A significant difference from uninfected mice was determined by ANOVA with Dunnett’s test for multiple comparisons on log-transformed data. ( **D**) Plasma concentrations of IFNɣ, TNFα, and IL-10 determined by Luminex multiplex bead array, with many falling below the limit of detection (12.4 pg/mL, 3.2 pg/mL, and 12.3 pg/mL, respectively). Data shown as box and whisker plot of 3-4 mice per time point. A significant difference from uninfected mice was determined by Dunn’s test with Bonferroni adjustment for multiple testing on log-transformed data, with (*) p<0.05, (**) p<0.01. Data for all mice are available in extended raw data.

In mice infected with blood stage recently mosquito-transmitted *P. chabaudi* AS parasites, expressing GFP ( rMT-PcAS-GFP), parasitaemia peaked 9 days post infection (p.i.) at a low to moderate density (median 1.32%, IQR 0.53-4.27, n=51) ( Figure 1), in line with expectations ( Spence *et al.*, 2013). There were no significant changes in weight compared to uninfected mice, but hemoglobin concentrations declined on day 11 p.i., as observed previously ( Marr *et al.*, 2020). There was a clear but very transient inflammatory response on day 7 p.i. (i.e. immediately prior to peak parasitaemia) characterized by raised plasma concentrations of IFN-ɣ and TNF-α. Plasma IL-10 concentrations also peaked on day 7 p.i. and were significantly raised for several days.

**Figure 2.**
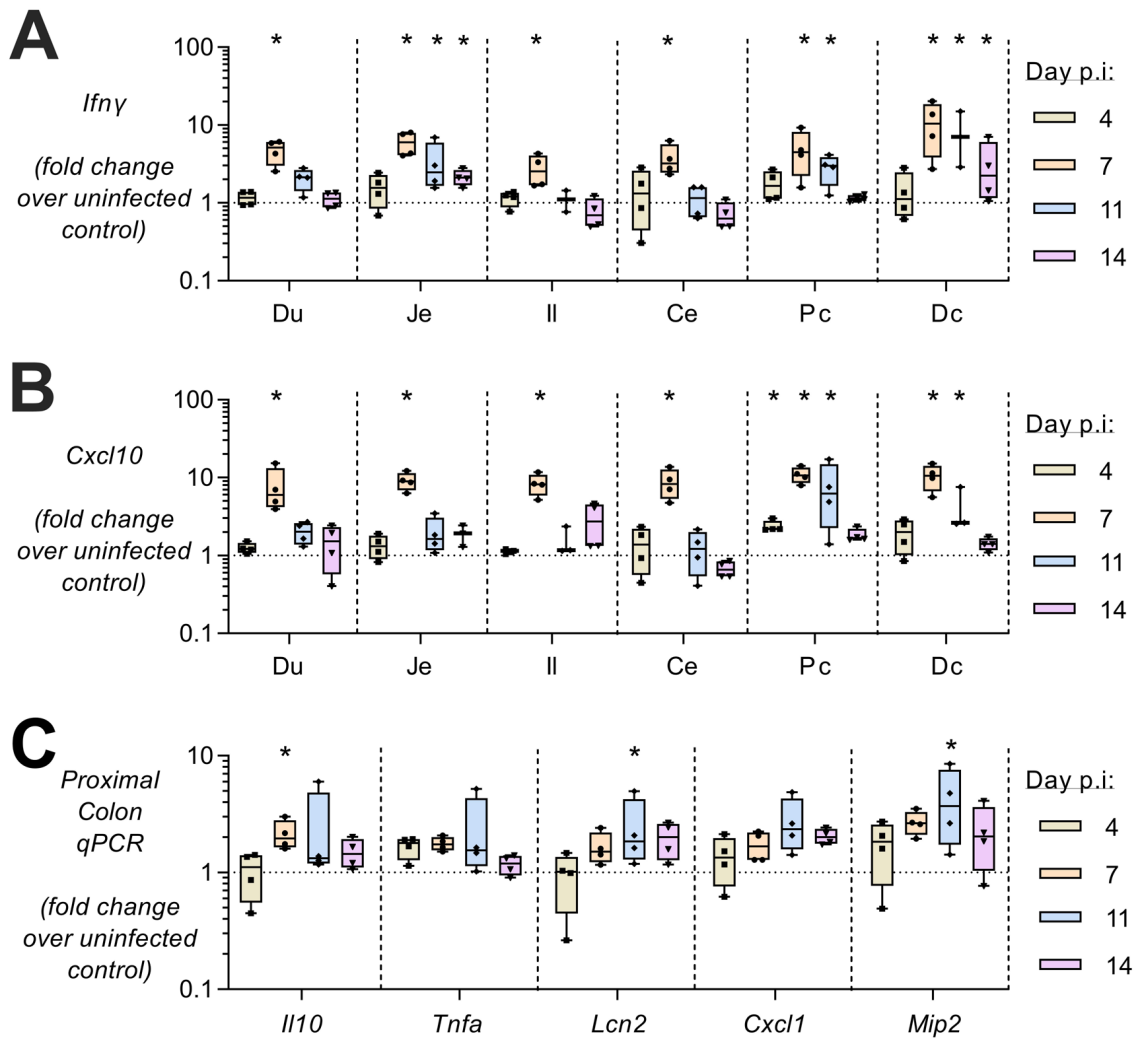
Intestinal inflammation during *P. chabaudi* infection in mice. At 4, 7, 11, and 14 days post infection with MT-PcAS-GFP, mice were culled at schizogony and intestines divided into 6 equal portions; duodenum (Du), jejunum (Je), ileum (Il), cecum (Ce), proximal colon (Pc), and distal colon (Dc). Transcript levels of inflammatory cytokines were determined from 1µg RNA by RT-PCR for ( **A**) *ifng*, and ( **B**) *cxcl10* in all tissue sections. ( **C**) In the proximal colon, the levels of *il10*, *tnfa*, *lcn2*, *cxcl1*, *mip2*, and *il27* were also determined. Data shown as fold change over uninfected control mice (with dotted line at 1, mean of n=25) for n=4 mice from Experiment 1 ( Figure 1A). Data shown as a box-whisker plot with dots representing individual mice, where a significant difference from uninfected mice was determined by ANOVA with Dunnett’s comparison test on log-transformed data, with (*) p<0.05. Data for all mice are available in extended raw data.

To determine whether rMT-PcAS-GFP infection and the associated systemic inflammatory response has any intestinal consequences, inflammatory markers were analysed in samples of duodenum, jejunum, ileum, caecum, proximal colon and distal colon by qRT-PCR ( Figure 2). Intestinal transcript levels for *ifng* and *cxcl10* were raised between 7 and 14 days p.i., and were significantly higher than controls in all sections of the intestine on day 7 p.i. Raised inflammatory markers were particularly evident in the proximal colon with significant elevations of *il10* on day 7 p.i., and *lcn2* and *mip2* at 11 days p.i. These data are indicative of a generalised, low-grade enteritis coincident with the period of peak parasitaemia.

**Figure 3.**
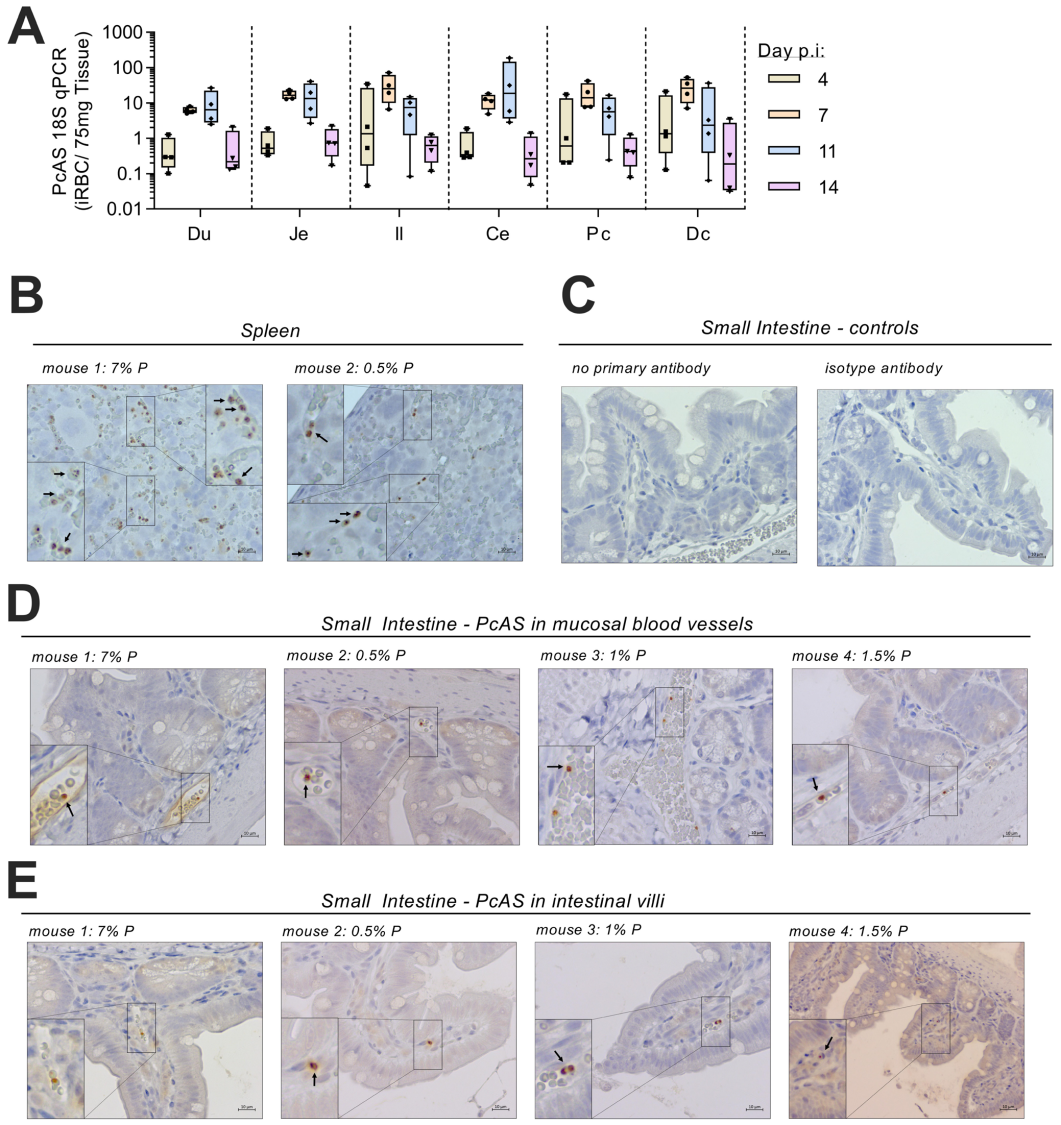
*P. chabaudi*-infected RBCs are present in the mouse intestine. ( **A**) At 4, 7, 11, and 14 days post infection with MT-PcAS-GFP, mice were culled at schizogony and intestines divided into 6 equal portions; duodenum (Du), jejunum (Je), ileum (Il), cecum (Ce), proximal colon (Pc), and distal colon (Dc). Concentrations of *P. chabaudi* r18S were determined by RT-PCR of 1µg of tissue RNA run in parallel with a standard of known iRBCs, and then normalised to 75mg of extracted tissue to account for differences between tissue sections. Data is representative of two independent experiments, with Experiment 1 ( Figure 1A) shown (n=4 per time point). Data for all mice are available in extended raw data. ( **B**– **E**) Immunohistochemistry of formalin-fixed, paraffin embedded intestinal rolls from Experiment 3 ( Figure 1A) at 7 d.p.i. ( Figure 1A). Tissues were treated with a primary antibody targeting GFP and developed using DAB substrate (brown pigment) with a counterstain of hematoxylin. Representative microscopy images from independent animals from ( **B**) spleen and ( **C–D**) small intestine, with parasites present in blood vessels of the ( **D**) mucosa and ( **E**) villi. Scale bar is 10µm. Additional full images, along with scans of slides by nanozoomer, available in extended raw data.

To determine whether parasite localization in the intestine may be driving the enteritis, qPCR for PcAS ribosomal 18S (r18s) was conducted on the same tissue samples ( Figure 3). PcAS r18s was detected in all sections of the intestine with the highest transcript levels detected on days 7 and 11 p.i. Mean intestinal PcAS r18s concentrations were highly correlated with peripheral parasitaemia on both day 7 p.i (r=0.84, p=0.008, n=8 from 2 independent experiments) and day 11 p.i. (r=0.99, p<0.0001, n=9 from 2 independent experiments).

As mice had not been perfused to remove intravascular blood prior to dissection, it was possible that parasites detected in the intestine were simply circulating in blood. However, we could not rule out the possibility that parasites might be sequestered within blood vessels or had entered the tissues ( Brugat *et al.*, 2014); cytoadherence of *P. chabaudi AS* via binding to the cell-surface receptor ICAM-1 has been reported in the spleen and liver ( Cunningham *et al.*, 2017). Therefore, to determine the tissue localization of intestinal rMT-PcAS-GFP, formalin-fixed tissue sections were analysed by immunohistochemistry ( Figure 3). Parasites in the spleen were a mixed population of rings, trophozoites and schizonts, as reported previously during schizogony of *P. chabaudi* AS ( Brugat *et al.*, 2014). By contrast, in the intestine, only trophozoites and ring forms were seen. Moreover, parasitised red cells in the intestine were clearly confined to intravascular spaces of mucosal blood vessels and smaller villous capillaries. These data suggest that, whilst iRBC circulate freely within intestinal vessels, there is no obvious indication of parasite sequestration in the intestine.

**Figure 4.**
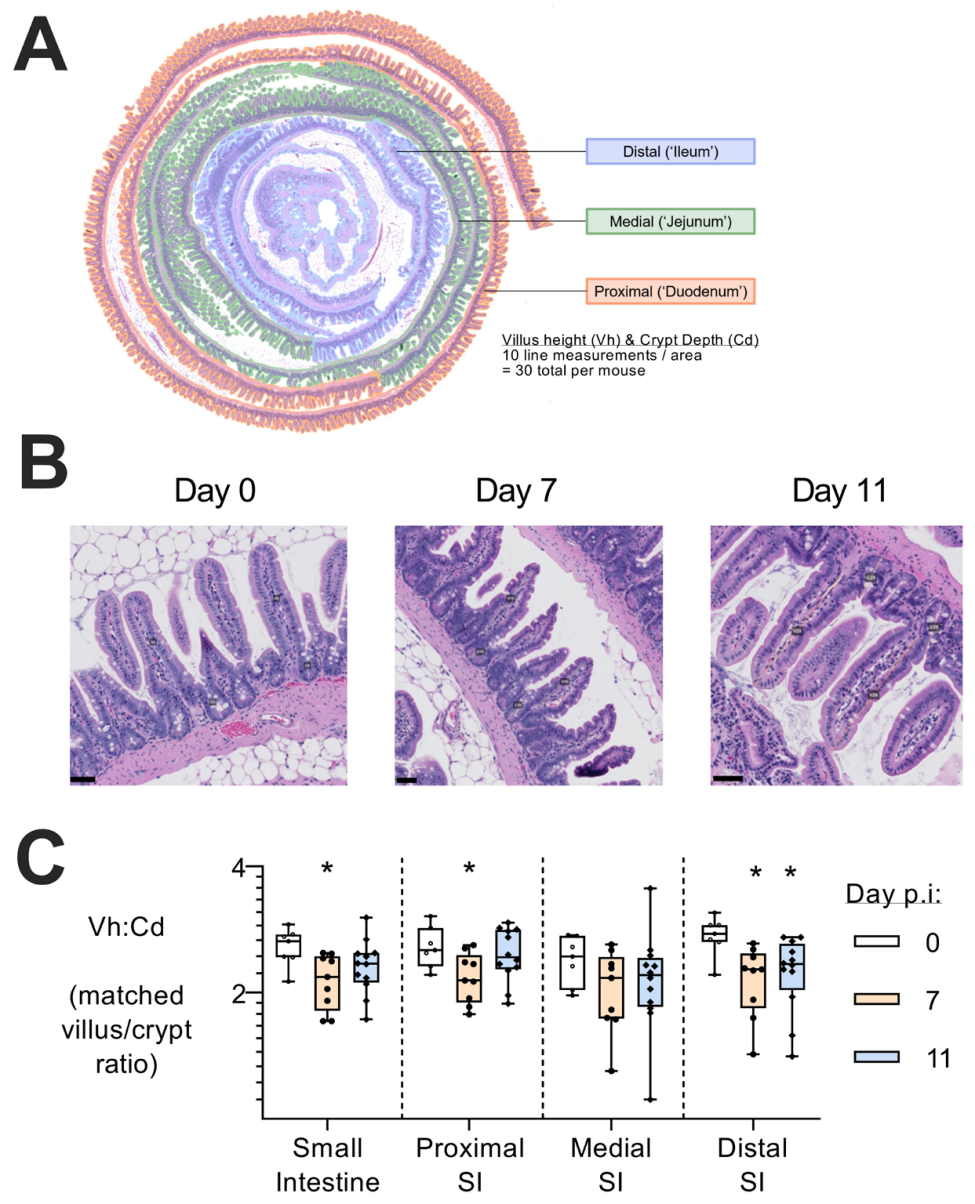
Morphological changes in the small intestine during *P. chabaudi* infection. ( **A**) Small intestinal tissue was rolled and formalin-fixed prior to staining with hematoxylin and eosin. Prepared slides were imaged with a nanozoomer, and subjectively divided into three section; proximal small intestine (SI) (considered the ‘duodenum’), medial SI (‘jejunum’), and distal SI (‘ileum’). Using blinded images on QuPath software, villus height:crypt depth ratios from 30 pairs of measurements per animal were measured (10 in proximal, distal, and medial), averaged, and displayed by box-whisker plot with dots representing individual mice. ( **B**) Representative images of histology images stained with hematoxylin and eosin, with annotated lines to measure villous height and crypt depth (Day 0 is slide #108, pairs 4 and 5; Day 7 is slide #69, pairs 18 and 19; Day 11 is slide #87, pairs 28 and 29). Scale bar = 50µM. Entire scans of slides by nanozoomer are available in extended electronic files, annotated with each measurement taken. ( **C**) Ratios of neighbouring villi height (Vh) and crypt depth (Cd) were calculated (n=30, as above) and then averaged per animal. Data measured from tissues taken from 2 experiments (Experiments 3 and 4, Figure 1A), uninfected (n=7), day 7 (n=9), and day 11 (n=12) post infection. Data shown as a box-whisker plot on log2 scale with dots representing individual mice, where a significant difference from uninfected mice was determined by ANOVA with Dunnett’s comparison test on log-transformed data, with (*) p<0.05.

Despite the lack of parasite sequestration in the intestine ( Figure 3), evidence of intestinal inflammation ( Figure 2) raised the possibility of morphological damage. Although there was no gross intestinal damage (villous epithelium was intact with no evidence of leucocyte infiltration, haemorrhage or necrosis), there was a significant reduction in villous height/crypt depth ratio across the small intestine at day 7 p.i. ( Figure 4), indicative of mild villous atrophy and/or crypt hyperplasia ( Mills, 2019).

Given the evidence of a generalized enteritis in PcAS-infected mice, we considered whether there might also be changes in intestinal permeability. For these experiments, mice were infected with recently mosquito-transmitted *P. chabaudi* AS expressing mCherry (rMT-PcAS-mCh) (in order not to interfere with measurement of FITC-dextran). To determine whether any changes in permeability might be linked to intestinal inflammation, faecal inflammatory protein concentrations were also assessed.

**Figure 5.**
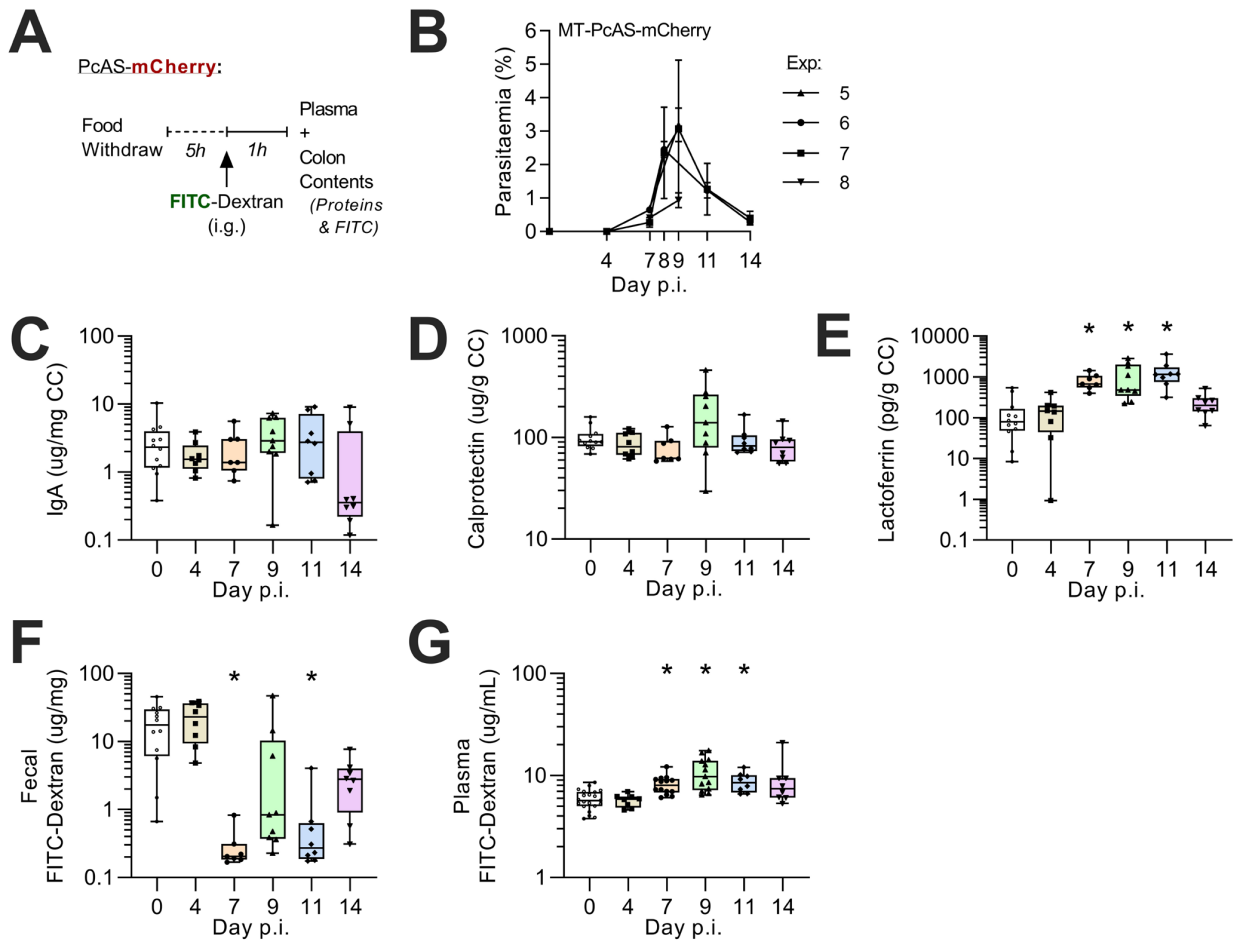
Increased membrane permeability and faecal lactoferrin during *P. chabaudi* infection. Female C57BL/6 mice were infected with 1x10 ^5^ rMT-PcAs-mCh-iRBCs. ( **A**) At various time-points, uninfected or rMT-PcAS-mCh infected mice were given FITC-dextran solution by oral gavage and culled 1 hour later. ( **B**) Prior to schizogony, peripheral parasitemia from tail blood from four independent experiments was determined by flow cytometry. Data shown as mean±SEM, n=4–5 per experiment. ( **C**– **E**) Colon content (CC) concentrations of ( **C**) immunoglobulin A (IgA), ( **D**) calprotectin, and ( **E**) lactoferrin were determined by ELISA from faecal homogenates. ( **F**) Fluorescence of FITC-dextran in faecal homogenates was measured. ( **G**) Plasma concentrations of FITC-dextran were determined. ( **C**– **G**) Data displayed as box-whisker plot with dots representing individual mice, n=7–12. Data combined from 2 experiments per time point, with additional mice for FITC-dextran ( **G**) from pilot data (Experiment #5 in panel 5B). A significant difference from uninfected mice was determined by ANOVA with Dunnett’s comparison test on log-transformed data, with (*) p<0.05.

**Figure 6.**
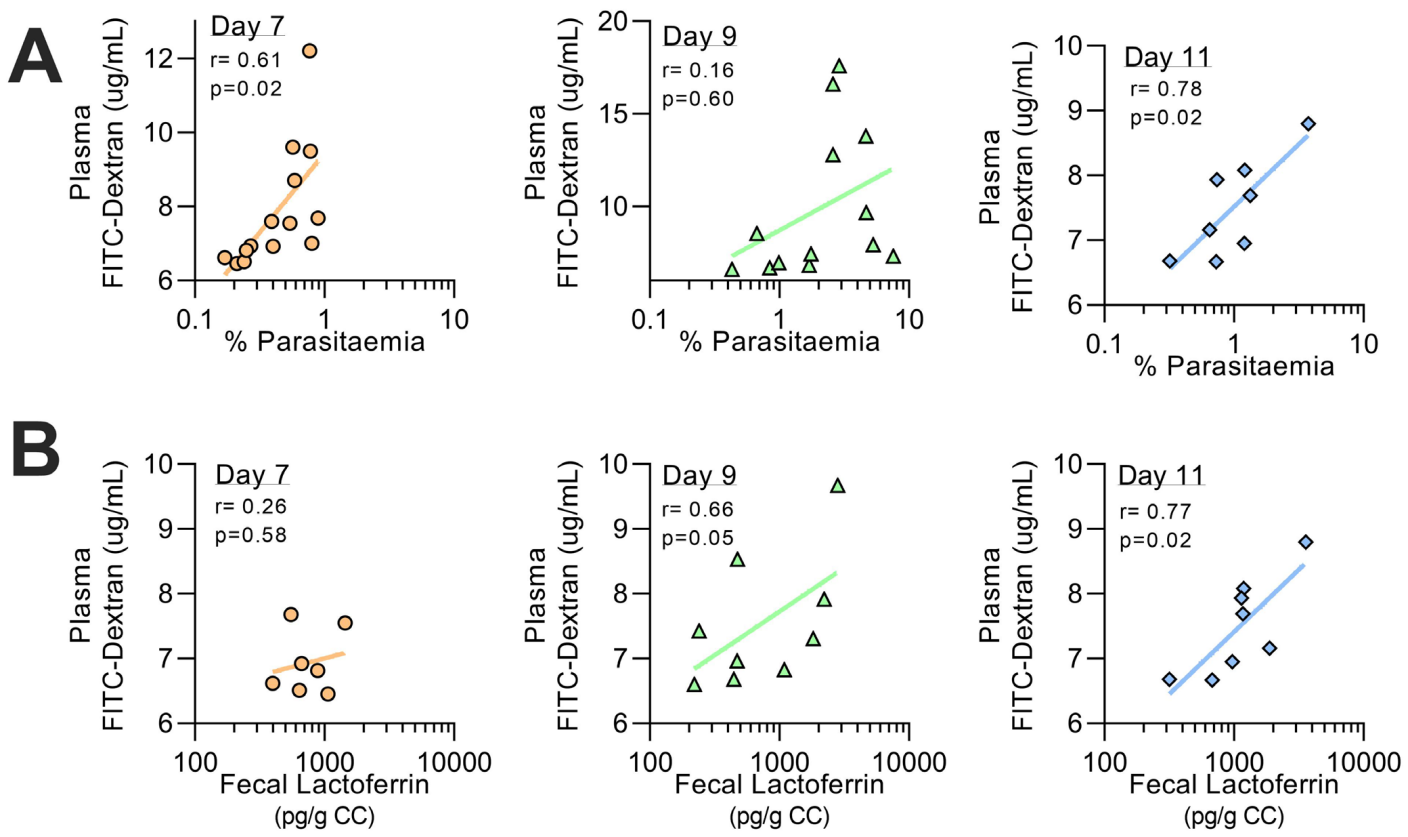
Relationship between parasitemia, faecal inflammation and intestinal permeability. Correlation analysis from mice infected with rMT-PcAS-mCh ( Figure 5B) from days 7, 9, and 11 p.i. was performed comparing permeability (plasma FITC-dextran) with ( **A**) peripheral parasitemia (%), prior to schizogony, and ( **B**) faecal calprotectin levels (pg per g of colon contents, CC). Data combined from 2 experiments per time point, with additional mice for panel (A) at day 7 and 9 from pilot data (Experiment #5 in Figure 5B) (n=7–14 mice per time point). Pearson correlation coefficient (r) and p value shown, along with fitted linear regression line.

Parasitaemia of rMT-PcAS-mCh peaked at a similar density to rMT-PcAS-GFP and between days 8–11 p.i. (median density 1.74%, IQR 0.86-4.67, n=21) ( Figure 5). Mice infected with rMT-PcAS-mCh were culled 1 hour after oral gavage with FITC-dextran ( Woting & Blaut, 2018), rather than after 4 hours as previously described ( Alamer *et al.*, 2019; Denny *et al.*, 2019; Taniguchi *et al.*, 2015). Faecal homogenates (colon contents) were analysed for FITC-dextran, secretory IgA and two biomarkers of intestinal inflammation, calprotectin and lactoferrin; ( Lamb & Mansfield, 2011). There was a trend for increased sIgA concentrations at peak parasitaemia (day 9 p.i.) but this did not reach statistical significance. However, faecal calprotectin concentrations were significantly raised at day 9 p.i. and faecal lactoferrin concentrations were significantly above baseline on days 9 and 11 p.i.. Furthermore, FITC-dextran concentrations were markedly and significantly lower in the faeces and higher in plasma 1 hour after oral administration on days 7 to 14 p.i. Of note, plasma FITC-dextran concentrations were highly correlated with peripheral parasite densities at day 7 p.i. (r=0.82, p<0.01) and day 11 p.i. (r=0.78, p=0.02) and with faecal lactoferrin concentrations on days 9 and 11 p.i. ( Figure 6). Taken together, these data suggest that both intestinal inflammation and increased intestinal permeability are secondary to circulating parasitaemia.

## Discussion

Using a rodent model of attenuated, resolving malaria (intraperitoneal injection of recently mosquito transmitted *P. chabaudi* AS) that more closely reflects mild to moderate human malaria infections, with rapidly resolving parasitaemia peaking below 2% and mild to moderate anaemia that resolves upon parasite clearance, we have confirmed previous reports of malaria-associated intestinal inflammation ( Alamer *et al.*, 2019; Mooney *et al.*, 2015; Shimada *et al.*, 2019; Taniguchi *et al.*, 2015) and significantly extended those observations. We have shown that parasite-iRBCs circulate freely in the intestinal vasculature but do not appear to sequester in this site; that the enteritis is generalized throughout the small and large intestines and coincident with the development and resolution of parasitaemia; and that intestinal permeability is markedly increased at the peak of parasitaemia and intestinal inflammation. This study has thus established a relevant murine model of malaria-associated enteritis that can be used to further our understanding of malarial disease and enteric co-infections.

Previous studies have looked for sequestration of *Plasmodium spp*-infected erythrocytes in the intestines. Using a luciferase tagged line of *P. chabaudi* AS, Brugat *et al.* (2014) observed parasites in the liver, spleen and lung but little if any luminescence from whole intestinal tissues ( Brugat *et al.*, 2014). *P. falciparum*-infected parasites have been identified in the small intestine, including the intestinal villi, at autopsy ( Pongponratn *et al.*, 1991; Seydel *et al.*, 2006) but the resolution of the images was insufficient to determine their precise anatomical localization. In our rMT-PcAS model, immunohistochemistry revealed that ring and trophozoite stage parasites were abundant in the mucosal and villous blood vessels but schizonts were not seen and there was no evidence of cytoadherence of iRBCs to the vascular endothelium or of infiltration of iRBCs into the extravascular spaces or deeper tissues.

Previous studies using murine malaria models that lead to high parasitaemia, severe anaemia and significant weight loss ( *P. yoelii ssp* and serially blood-passaged *P. chabaudi*) have reported moderate intestinal inflammation, with infiltration of the intestinal mucosa by monocytes, mast cells, and T cells ( Alamer *et al.*, 2019; Chau *et al.*, 2013; Mooney *et al.*, 2015), epithelial damage ( Mooney *et al.*, 2015) and villous atrophy and haemorrhages ( Taniguchi *et al.*, 2015). By contrast, rMT-PcAS induced enteritis was much more subtle with modestly reduced villous/crypt ratios but no gross epithelial damage, inflammation or haemorrhage. Overall, however, it seems that the severity of malaria-associated enteritis reflects the severity of the infection *per se*. This, taken together with the lack of evidence for sequestration of *P. chabaudi*-parasitised erythrocytes in the intestine and the close temporal correlation between enteritis and circulating parasite density (parasitaemia), suggests that the enteritis may be driven by systemic inflammation rather than localization of parasitised RBCs in the intestine.

Although the enteritis observed during rMT-PcAS infection was relatively mild, it was sufficient to cause a marked increase in intestinal permeability during the period of peak parasitaemia, as evidenced by very rapid translocation of FITC-dextran from the gut lumen (colon contents) into the plasma. Moreover, this increased permeability was highly correlated with both parasitaemia and intestinal inflammation (faecal calprotectin), suggesting a causal pathway in which parasitaemia drives systemic inflammation, systemic inflammation drives enteric inflammation and enteric inflammation drives increased intestinal permeability.

Increased intestinal permeability has been demonstrated in humans infected with *P. falciparum* ( Pongponratn *et al.*, 1991) and in *P. yoelii nigeriensis-infected* mice ( Chau *et al.*, 2013)) (in both cases using the lactulose mannitol test ( FlemIng *et al.*, 1990)) and in more *P. berghei* ANKA, *Plasmodium yoelii* 17XNL, and PcAS models (using FITC-dextran) ( Alamer *et al.*, 2019; Denny *et al.*, 2019; Taniguchi *et al.*, 2015), with varying degrees of increased permeability observed either early or at the peak of infection. The consequences of this change in intestinal permeability, especially in terms of maintenance of the barrier function of the intestinal epithelium and risk of translocation, invasion and systemic dissemination of enteric pathogens such as NTS ( Mooney *et al.*, 2019), remain to be discerned.

In summary, we have established a clinically relevant murine model of malaria-associated enteritis characterized by systemic and local inflammation and increased intestinal permeability. This model can be exploited to better understand the pathophysiology of enteric disease during malaria infections and to understand the mechanisms by which current or recent malaria infections substantially increase the risk of invasive enteric bacterial infections.

## Data availability

### Underlying data

Open Science Framework: Malaria in the Murine Intestine, https://doi.org/10.17605/OSF.IO/EDQTK. ( Mooney, 2022a)

This project contains the following underlying data:

- Methods - Animal Numbers.xlsx
- Fig 1 - Anemia by Hemocue.xlsx
- Fig 1 - Parasitemia_PcAS-GFP.xlsx
- Fig 1 - Plasma Protein by Luminex.xlsx
- Weight Change.xlsx
- Mouse Luminex Raw File.rbx
- Parasitemia FACS Files – Raw and Analysed
- Fig 2 - Inflammation qPCR in Tissue.xlsx
- qPCR Raw Files.xlsx
- 18S PCR Raw Files
- Fig 3 - 18S Plasmodium qPCR in Tissue.xlsx
- Histology Images.jpg
- H'n'E Inventory and Quality Scoring _ Analysed JM 16 Aug.xlsx
- QuPath Project SI.zip
- FACS Raw Files
- Fecal Supe ELISAs.xlsx
- Fecal Supe FITC.xlsx
- FITC-Dextran Plasma RFU.xlsx
- Parasitemia-PcAS mCherry.xlsx
- mCherry correlation data.xlsx

Edinburgh Datashare: Malaria in the Murine Intestine, https://doi.org/10.7488/ds/3434. ( Mooney, 2022b)

This project contains the following underlying data:

- Figure3-IHC_for_malaria_in_Small_Intestine.zip
- Figure4-HnE_of_Small_Intestine.zip

### Reporting guidelines

Open Science Framework: ARRIVE checklist for “Intestinal inflammation and increased intestinal permeability in Plasmodium chabaudi AS infected mice”, https://doi.org/10.17605/OSF.IO/EDQTK. ( Mooney, 2022a)

Data are available under the terms of the Creative Commons Attribution 4.0 International license (CC-BY 4.0).

## References

[ref-1] Alamer E Carpio VH Ibitokou SA : Dissemination of non-typhoidal *Salmonella* during *Plasmodium chabaudi* infection affects anti-malarial immunity. *Parasitol Res.* 2019;118(7):2277–2285. 10.1007/s00436-019-06349-z 31119381 PMC6686885

[ref-2] Bankhead P Loughrey MB Fernández JA : QuPath: Open source software for digital pathology image analysis. *Sci Rep.* 2017;7(1):16878. 10.1038/s41598-017-17204-5 29203879 PMC5715110

[ref-3] Biggs HM Lester R Nadjm B : Invasive *Salmonella* infections in areas of high and low malaria transmission intensity in Tanzania. *Clin Infect Dis.* 2014;58(5):638–47. 10.1093/cid/cit798 24336909 PMC3922215

[ref-4] Brugat T Cunningham D Sodenkamp J : Sequestration and histopathology in *Plasmodium chabaudi* malaria are influenced by the immune response in an organ-specific manner. *Cell Microbiol.* 2014;16(5):687–700. 10.1111/cmi.12212 24003897 PMC4234010

[ref-5] Chau JY Tiffany CM Nimishakavi S : Malaria-Associated L-Arginine Deficiency Induces Mast Cell-Associated Disruption to Intestinal Barrier Defenses against Nontyphoidal *Salmonella Bacteremia*. *Infect Immun.* 2013;81(10):3515–26. 10.1128/IAI.00380-13 23690397 PMC3811760

[ref-6] Cunningham DA Lin JW Brugat T : ICAM-1 is a key receptor mediating cytoadherence and pathology in the *Plasmodium chabaudi* malaria model. *Malar J.* 2017;16(1):185. 10.1186/s12936-017-1834-8 28468674 PMC5415785

[ref-7] Denny JE Powers JB Castro HF : Differential sensitivity to *plasmodium yoelii* infection in C57BL/6 mice impacts gut-liver axis homeostasis. *Sci Rep.* 2019;9(1):3472. 10.1038/s41598-019-40266-6 30837607 PMC6401097

[ref-8] Dudgeon LS Clarke C : An Investigation on Fatal Cases of Pernicious Malaria Caused by *Plasmodium Falciparum* in Macedonia. *QJM: An International Journal of Medicine.* 1919;os-12(48):372–390. 10.1093/qjmed/os-12.48.372

[ref-9] Fidler G Tolnai E Stagel A : Tendentious effects of automated and manual metagenomic DNA purification protocols on broiler gut microbiome taxonomic profiling. *Sci Rep.* 2020;10(1):3419. 10.1038/s41598-020-60304-y 32099013 PMC7042355

[ref-10] Fleming SC Kapembwa MS Laker MF : Rapid and simultaneous determination of lactulose and mannitol in urine, by HPLC with pulsed amperometric detection, for use in studies of intestinal permeability. *Clin Chem.* 1990;36(5):797–9. 10.1093/clinchem/36.5.797 2110873

[ref-11] Geus D Sifft KC Habarugira F : Co-infections with *Plasmodium, Ascaris* and *Giardia* among Rwandan schoolchildren. *Trop Med Int Health.* 2019;24(4):409–420. 10.1111/tmi.13206 30659700

[ref-12] Karney WW Tong MJ : Malabsorption in *Plasmodium Falciparum* Malaria. *Am J Trop Med Hyg.* 1972;21(2):1–5. 10.4269/ajtmh.1972.21.1 4550149

[ref-13] Lamb CA Mansfield JC : Measurement of faecal calprotectin and lactoferrin in inflammatory bowel disease. *Frontline Gastroenterol.* 2011;2(1):13–18. 10.1136/fg.2010.001362 23904968 PMC3724198

[ref-14] Lo Vecchio A Basile FW Bruzzese D : Diarrhea in Children with *Plasmodium falciparum* Malaria: A Case-Control Study on the Prevalence and Response to Antimalarial Treatment. *Am J Trop Med Hyg.* 2021;104(2):659–665. 10.4269/ajtmh.20-0287 33319726 PMC7866346

[ref-15] Lokken KL Mooney JP Butler BP : Malaria parasite infection compromises control of concurrent systemic non-typhoidal *Salmonella* infection via IL-10-mediated alteration of myeloid cell function. *PLoS Pathog.* 2014;10(5):e1004049. 10.1371/journal.ppat.1004049 24787713 PMC4006898

[ref-16] Marr EJ Milne RM Anar B : An enhanced toolkit for the generation of knockout and marker-free fluorescent *Plasmodium chabaudi* [version 2; peer review: 2 approved]. *Wellcome Open Res.* 2020;5(71):71. 10.12688/wellcomeopenres.15587.2 32500098 PMC7236590

[ref-17] Mills S : Histology for pathologists.Lippincott Williams & Wilkins,2019. Reference Source

[ref-18] Mooney JP : Malaria in the Murine Intestine, [dataset]. *Open Science Framework.* 2022a. 10.17605/OSF.IO/EDQTK

[ref-19] Mooney JP Mooney JP : Malaria in the Murine Intestine, [dataset].University of Edinburgh. School of Biological Sciences.2022b. 10.7488/ds/3434

[ref-20] Mooney JP Galloway LJ Riley EM : Malaria, anemia, and invasive bacterial disease: A neutrophil problem? *J Leukoc Biol.* 2019;105(4):645–655. 10.1002/JLB.3RI1018-400R 30570786 PMC6487965

[ref-21] Mooney JP Lokken KL Byndloss MX : Inflammation-associated alterations to the intestinal microbiota reduce colonization resistance against non-typhoidal *Salmonella* during concurrent malaria parasite infection. *Sci Rep.* 2015;5:14603. 10.1038/srep14603 26434367 PMC4592952

[ref-22] Organization W. H: World malaria report 2021.2021. Reference Source

[ref-23] Park SE Pak GD Aaby P : The Relationship Between Invasive Nontyphoidal *Salmonella* Disease, Other Bacterial Bloodstream Infections, and Malaria in Sub-Saharan Africa. *Clin Infect Dis.* 2016;62 Suppl 1(Suppl 1):S23–S31. 10.1093/cid/civ893 26933016 PMC4772835

[ref-24] Pongponratn E Riganti M Punpoowong B : Microvascular Sequestration of Parasitized Erythrocytes in Human Falciparum Malaria: a Pathological Study. *Am J Trop Med Hyg.* 1991;44(2):168–175. 10.4269/ajtmh.1991.44.168 2012260

[ref-25] Scott JA Berkley JA Mwangi I : Relation between falciparum malaria and bacteraemia in Kenyan children: a population-based, case-control study and a longitudinal study. *Lancet.* 2011;378(9799):1316–1323. 10.1016/S0140-6736(11)60888-X 21903251 PMC3192903

[ref-26] Sey ICM Ehimiyein AM Bottomley C : Does Malaria Cause Diarrhoea? A Systematic Review. *Front Med (Lausanne).* 2020;7(703):589379. 10.3389/fmed.2020.589379 33330549 PMC7717985

[ref-27] Seydel KB Milner DA Jr Kamiza SB : The distribution and intensity of parasite sequestration in comatose Malawian children. *J Infect Dis.* 2006;194(2):208–215. 10.1086/505078 16779727 PMC1515074

[ref-28] Shimada M Hirose Y Shimizu K : Upper gastrointestinal pathophysiology due to mouse malaria *Plasmodium berghei* ANKA infection. *Trop Med Health.* 2019;47(1):18. 10.1186/s41182-019-0146-9 30872946 PMC6399856

[ref-29] Sowunmi A Ogundahunsi OA Falade CO : Gastrointestinal manifestations of acute falciparum malaria in children. *Acta Trop.* 2000;74(1):73–76. 10.1016/s0001-706x(99)00043-1 10643910

[ref-30] Spence PJ Brugat T Langhorne J : Mosquitoes Reset Malaria Parasites. *PLoS Pathog.* 2015;11(7):e1004987. 10.1371/journal.ppat.1004987 26133171 PMC4489640

[ref-31] Spence PJ Jarra W Lévy P : Vector transmission regulates immune control of *Plasmodium* virulence. *Nature.* 2013;498(7453):228–231. 10.1038/nature12231 23719378 PMC3784817

[ref-32] Stresman G Sepúlveda N Fornace K : Association between the proportion of *Plasmodium falciparum* and *Plasmodium vivax* infections detected by passive surveillance and the magnitude of the asymptomatic reservoir in the community: a pooled analysis of paired health facility and community data. *Lancet Infect Dis.* 2020;20(8):953–963. 10.1016/S1473-3099(20)30059-1 32277908 PMC7391005

[ref-33] Takem EN Roca A Cunnington A : The association between malaria and non-typhoid Salmonella bacteraemia in children in sub-Saharan Africa: a literature review. *Malar J.* 2014;13:400. 10.1186/1475-2875-13-400 25311375 PMC4210537

[ref-34] Taniguchi T Miyauchi E Nakamura S : *Plasmodium berghei* ANKA causes intestinal malaria associated with dysbiosis. *Sci Rep.* 2015;5:15699. 10.1038/srep15699 26503461 PMC4621605

[ref-35] Troeger C Blacker BF Khalil IA : Estimates of the global, regional, and national morbidity, mortality, and aetiologies of diarrhoea in 195 countries: a systematic analysis for the Global Burden of Disease Study 2016. *Lancet Infect Dis.* 2018;18(11):1211–1228. 10.1016/S1473-3099(18)30362-1 30243583 PMC6202444

[ref-36] Volynets V Reichold A Bárdos G : Assessment of the Intestinal Barrier with Five Different Permeability Tests in Healthy C57BL/6J and BALB/cJ Mice. *Dig Dis Sci.* 2016;61(3):737–746. 10.1007/s10620-015-3935-y 26520109

[ref-37] Woting A Blaut M : Small Intestinal Permeability and Gut-Transit Time Determined with Low and High Molecular Weight Fluorescein Isothiocyanate-Dextrans in C3H Mice. *Nutrients.* 2018;10(6):685. 10.3390/nu10060685 29843428 PMC6024777

