# Supplementary material for: Intestinal inflammation and increased intestinal permeability in Plasmodium chabaudi AS infected mice
Source: Wellcome Open Res. 2022 Oct 5;7:134. Originally published 2022 Apr 13. [Version 2] doi: 10.12688/wellcomeopenres.17781.2 (PMC9647155; doi:10.12688/wellcomeopenres.17781.2)
Supplement: Peer review history

# Peer review: Reviewer response for version 2

**DOI:** 10.21956/wellcomeopenres.20398.r52675

**Published:** 2022-11-09

**Authors:** Kevin Maloy

**Affiliations:** 1 School of Infection and Immunity, College of Medical, Veterinary and Life Sciences, University of Glasgow, Glasgow, UK

I thank the authors for having comprehensively addressed my comments and suggestions. The revised manuscript represents a solid addition to this field and I am happy to approve version 2.

Is the work clearly and accurately presented and does it cite the current literature?

Yes

If applicable, is the statistical analysis and its interpretation appropriate?

Yes

Are all the source data underlying the results available to ensure full reproducibility?

Yes

Is the study design appropriate and is the work technically sound?

Yes

Are the conclusions drawn adequately supported by the results?

Yes

Are sufficient details of methods and analysis provided to allow replication by others?

Yes

Reviewer Expertise:

Mucosal Immunology

I confirm that I have read this submission and believe that I have an appropriate level of expertise to confirm that it is of an acceptable scientific standard.

# Peer review: Reviewer response for version 2

**DOI:** 10.21956/wellcomeopenres.20398.r52674

**Published:** 2022-10-12

**Authors:** Rahul Vijay

**Affiliations:** 1 Center for Cancer Cell Biology, Immunology and Infection, Rosalind Franklin University, Chicago, IL, USA; 2 Rosalind Franklin University of Medicine and Science, Chicago, IL, USA

This reviewer does not have any additional comments to the manuscript.

Is the work clearly and accurately presented and does it cite the current literature?

Yes

If applicable, is the statistical analysis and its interpretation appropriate?

I cannot comment. A qualified statistician is required.

Are all the source data underlying the results available to ensure full reproducibility?

Yes

Is the study design appropriate and is the work technically sound?

Partly

Are the conclusions drawn adequately supported by the results?

Partly

Are sufficient details of methods and analysis provided to allow replication by others?

Yes

Reviewer Expertise:

Immunology, parasitology

I confirm that I have read this submission and believe that I have an appropriate level of expertise to confirm that it is of an acceptable scientific standard.

# Peer review: Reviewer response for version 1

**DOI:** 10.21956/wellcomeopenres.19680.r51410

**Published:** 2022-07-25

**Authors:** Rahul Vijay

**Affiliations:** 1 Center for Cancer Cell Biology, Immunology and Infection, Rosalind Franklin University, Chicago, IL, USA; 2 Rosalind Franklin University of Medicine and Science, Chicago, IL, USA

In this study the authors report that gastrointestinal symptoms noted during malaria are indeed associated with the malarial disease and are not just coincidental. Using blood stage recently mosquito-transmitted *P. chabaudi* AS, they show the presence of low-grade intestinal inflammation and increased intestinal permeability that is concomitant with peak parasitemia. While parasite sequestration in the intestine is not observed, infected RBCs were seen circulating in the intestinal vasculature.

 As noted by the authors similar observations have been made in other mouse models of malaria but these observations, they claim, seem to better represent what is normally observed during human malaria in endemic regions.

 Major points:

- While assessing transcripts levels of various cytokines in the intestine, the authors have grouped *il10 *along with the inflammatory markers. As the authors may be very well aware, IL10 is considered anti-inflammatory cytokine and hence they need to make that clear in the text. It is also recommended that they give a brief description as to the implications of both pro and anti-inflammatory cytokines upregulated simultaneously.

Minor points:

- The study is merely observational without any mechanistic experimentation. Despite this, the authors have attempted to draw parallels between intestinal complications arising from human malarial disease and those that they observed in this study (using this particular mouse model of malaria), thus validating why their study more representative and useful for experimentation to learn that particular aspect during malaria.

Is the work clearly and accurately presented and does it cite the current literature?

Yes

If applicable, is the statistical analysis and its interpretation appropriate?

I cannot comment. A qualified statistician is required.

Are all the source data underlying the results available to ensure full reproducibility?

Yes

Is the study design appropriate and is the work technically sound?

Partly

Are the conclusions drawn adequately supported by the results?

Partly

Are sufficient details of methods and analysis provided to allow replication by others?

Yes

Reviewer Expertise:

Immunology, parasitology

I confirm that I have read this submission and believe that I have an appropriate level of expertise to confirm that it is of an acceptable scientific standard, however I have significant reservations, as outlined above.

# Response

**Published:** 2022-09-14

**Authors:** Jason Mooney

**Affiliations:** University of Edinburgh, UK

In this study the authors report that gastrointestinal symptoms noted during malaria are indeed associated with the malarial disease and are not just coincidental. Using blood stage recently mosquito-transmitted *P. chabaudi* AS, they show the presence of low-grade intestinal inflammation and increased intestinal permeability that is concomitant with peak parasitemia. While parasite sequestration in the intestine is not observed, infected RBCs were seen circulating in the intestinal vasculature.

 As noted by the authors similar observations have been made in other mouse models of malaria but these observations, they claim, seem to better represent what is normally observed during human malaria in endemic regions.

 Major points:

 While assessing transcripts levels of various cytokines in the intestine, the authors have grouped il10 along with the inflammatory markers. As the authors may be very well aware, IL10 is considered anti-inflammatory cytokine and hence they need to make that clear in the text. It is also recommended that they give a brief description as to the implications of both pro and anti-inflammatory cytokines upregulated simultaneously.

**----**

**RESPONSE:**

 Thank you for your comments and for this observation. The simultaneous production of both pro- and anti-inflammatory cytokines is well noted, and its purpose is to protect the host from immune-mediated pathology. In fact, previous work from co-author Eleanor Riley and others have shown that, in a highly virulent *P. yoelii* malaria model, Th1 cells are a major source of both IL-10 and IFNγ+ (Couper et al. 2008, DOI: 10.1371/journal.ppat.1000004). This work was then extended by Rosario et al. (2012) (DOI: 10.4049/jimmunol.1102755) during virulent *P. chabaudi* infection using IL-10 reporter mice. Therefore, we have now included additional clarification in the results section. Thank you for the opportunity for us to provide additional context to this phenomenon for the reader.

*Updated text:*

 Results (paragraph 1):

 “These kinetics are typical of acute malaria parasite infections, denoting a switch from a pro- to anti-inflammatory systemic response to protect the host from immunopathology, driven in part by the co-production of IFNɣ+ and IL-10+ in activated T cells ((Couper, Blount et al. 2008, Couper, Blount et al. 2008, do Rosário, Lamb et al. 2012)).”

**----**

 Minor points:

 The study is merely observational without any mechanistic experimentation. Despite this, the authors have attempted to draw parallels between intestinal complications arising from human malarial disease and those that they observed in this study (using this particular mouse model of malaria), thus validating why their study more representative and useful for experimentation to learn that particular aspect during malaria.

**----**

**RESPONSE:**

 Thank you for your point and we agree this is entirely observational work. We hope to utilize this mouse model, along with extending to human malaria, to address both the clinical relevance and underlying mechanism(s). We have clearly defined this work as observational and further commented on future mechanistic work in the discussion.

*Updated text:*

 Discussion (paragraph 6):

 “This study reveals novel observational changes in the intestine during a mild, avirulent murine malaria parasite infection. Future work is needed to unpick the mechanism by which intestinal inflammation is induced, and how this relates to intestinal permeability. This would include phenotypical analysis of the cellular source of the inflammatory mediators observed (e.g. IFN-γ, TNF-α, lactoferrin), as well as their location within the tissue and their contribution to intestinal permeability.”

**----**

# Peer review: Reviewer response for version 1

**DOI:** 10.21956/wellcomeopenres.19680.r49962

**Published:** 2022-06-15

**Authors:** Kevin Maloy

**Affiliations:** 1 School of Infection and Immunity, College of Medical, Veterinary and Life Sciences, University of Glasgow, Glasgow, UK

This study characterizes intestinal pathology in a well characterized murine model of malaria infection. This is an important topic because human malaria patients often exhibit gastrointestinal symptoms, especially diarrhoea, and they are also more prone to develop invasive disease associated with non-typhoidal Salmonella infection. Determining the contribution of malaria infection versus coincidental enteric infections to intestinal pathology is difficult, so mouse models of malaria are useful in this context. There have been reports of intestinal pathology in mouse models of severe malaria, but the extent of intestinal alterations in milder rodent malaria infections (that mimic the majority of infections in endemic areas) had not been investigated. Hence, this study employed infection with ‘recently mosquito-transmitted’ *Plasmodium chabaudi* AS (PcAS) and assessed intestinal pathology.

 Figure 1 presents data showing that the PcAS infection elicited a consistent pattern of low-to-moderate parasitaemia that peaked around day 9 post-infection (pi). Further analyses of one experiment indicated that there was a transient systemic inflammatory cytokine response on day 7 pi. It would be helpful to know if this pattern of systemic cytokine release was reproducible across multiple experiments.

 Figure 2A and B demonstrate that PcAS infection also led to increased expression of *ifng* and *cxcl10* along the entire intestinal tract and that these peaked again at day 7 pi. Figure 2C extended the analyses to additional markers for the proximal colon samples. Again, it would be helpful to know that these changes were reproducible across multiple experiments. In addition, whether the changes in gene expression corresponded to increased mediator secretion could have been tested using intestinal explants followed by Luminex and/or ELISA assays of the supernatants.

 Figure 4 presents histological analyses of changes in small intestinal morphology in PcAS-infected mice. They observed a modest, transient reduction in villus/crypt ratio on day 7 pi and infer that this reflects ‘mild villous atrophy and or crypt hyperplasia’. It should be possible to discern these by separately plotting the villous and crypt lengths. In addition, it would be of interest to know whether the composition and/or activation status of leukocyte populations in the lamina propria was affected by PcAs infection, although this is perhaps a question for future studies.

 Figure 5 presents data showing that PcAS infection also led to increased intestinal permeability, as evidenced by increased levels of plasma FITC-dextran, as well as elevated lactoferrin in the colon contents. Figure 6 extends these analyses by showing that these increases correlated with the levels of parasitaemia, suggesting that they are a consequence of the systemic infection. In this respect, it would be of interest to determine whether plasma IFNg and TNF levels also correlated positively with the measures of intestinal permeability.

 Overall, the data are clear, well controlled and very nicely presented. The conclusions made by the investigators are balanced and reasonable and the discussion places them into the wider context and identifies areas for further study.

 The key strengths of the study are that it represents a good model of milder malaria infection and presents clear data and detailed kinetic analyses. The model offers scope for further development to better understand the consequences of altered intestinal permeability on the ongoing malaria infection, as well as predisposition to enteric pathogens.

 The limitations are that the data are largely descriptive and offer limited mechanistic insight. For example, which cytokines are driving the increased intestinal permeability and what are the key cellular sources of these inflammatory mediators? Are they all systemic or are some local to the intestine? The model provides a solid basis for interrogating these and other questions.

Is the work clearly and accurately presented and does it cite the current literature?

Yes

If applicable, is the statistical analysis and its interpretation appropriate?

Yes

Are all the source data underlying the results available to ensure full reproducibility?

Yes

Is the study design appropriate and is the work technically sound?

Yes

Are the conclusions drawn adequately supported by the results?

Yes

Are sufficient details of methods and analysis provided to allow replication by others?

Yes

Reviewer Expertise:

Mucosal Immunology

I confirm that I have read this submission and believe that I have an appropriate level of expertise to confirm that it is of an acceptable scientific standard, however I have significant reservations, as outlined above.

# Response

**Published:** 2022-09-14

**Authors:** Jason Mooney

**Affiliations:** University of Edinburgh, UK

This study characterizes intestinal pathology in a well characterized murine model of malaria infection. This is an important topic because human malaria patients often exhibit gastrointestinal symptoms, especially diarrhoea, and they are also more prone to develop invasive disease associated with non-typhoidal Salmonella infection. Determining the contribution of malaria infection versus coincidental enteric infections to intestinal pathology is difficult, so mouse models of malaria are useful in this context. There have been reports of intestinal pathology in mouse models of severe malaria, but the extent of intestinal alterations in milder rodent malaria infections (that mimic the majority of infections in endemic areas) had not been investigated. Hence, this study employed infection with ‘recently mosquito-transmitted’ Plasmodium chabaudi AS (PcAS) and assessed intestinal pathology.

 Figure 1 presents data showing that the PcAS infection elicited a consistent pattern of low-to-moderate parasitaemia that peaked around day 9 post-infection (pi). Further analyses of one experiment indicated that there was a transient systemic inflammatory cytokine response on day 7 pi. It would be helpful to know if this pattern of systemic cytokine release was reproducible across multiple experiments.

**----**

**RESPONSE**:

 Thank you. This is a very valid point. Previously, we showed data from a representative experiment for clarity in figures 1-4, as the data showed similar conclusions across independent experiments. In response to your comment, and for uniformity, all graphs in this updated manuscript version now show pooled data from at least three independent experiments ( *including Fig 2A-C, Fig 3A, and Fig 4*).  As with all WOR publications, raw data is open and accessible for full scrutiny, and version 1, with the representative data, remains available online.

**----**

 Figure 2A and B demonstrate that PcAS infection also led to increased expression of ifng and cxcl10 along the entire intestinal tract and that these peaked again at day 7 pi. Figure 2C extended the analyses to additional markers for the proximal colon samples. Again, it would be helpful to know that these changes were reproducible across multiple experiments. In addition, whether the changes in gene expression corresponded to increased mediator secretion could have been tested using intestinal explants followed by Luminex and/or ELISA assays of the supernatants.

**----**

**RESPONSE**:

 (Comment 1) As outlined above, pooled data from two independent experiments is now shown.

 (Comment 2) We agree that directly testing for a correlation between mRNA and inflammatory protein expression within tissues, either by explant culture and/or FISH, would be definitive experiments. Here, we have asked whether circulating IFNγ correlates to intestinal transcripts, and have graphed the transcript level of *Ifnγ*, in both the proximal and distal colon, against circulating plasma levels in new Figure 2B. At 7 d.p.i, when circulating IFNγ is highest, there is a strong positive correlation. As Th1 cells are a major source of IFNγ in PcAS infection, this isn’t unexpected. However, at 11 d.p.i, when circulating IFNγ has significantly diminished, *Ifnγ* transcripts remain in the colon. This data suggests that IFNγ may come from both local and systemic sources, depending on the infection dynamics and we have discussed this conclusion further in paragraph 1 of the results. Indeed, dissecting out the cellular sources, along with their location within the tissue (i.e. in the blood vessels and/or lamina propria) would be important in future. Thank you for the thoughtful comment.

*Updated text for ‘Comment 2’:*

 Results (paragraph 2):

 “Whilst plasma IFNɣ peaks at 7 days p.i., intestinal transcripts of ifng remain elevated in the colon through to day 11 p.i., and show a significant positive correlation to the circulating protein levels in the proximal colon, but not the distal colon. Thus, ifng transcripts can remain in the intestine whilst circulating protein levels have become undetectable. These data are indicative of a generalised, low-grade enteritis which coincides with the period of peak parasitaemia and systemic inflammatory response.”

**----**

 Figure 4 presents histological analyses of changes in small intestinal morphology in PcAS-infected mice. They observed a modest, transient reduction in villus/crypt ratio on day 7 pi and infer that this reflects ‘mild villous atrophy and or crypt hyperplasia’. It should be possible to discern these by separately plotting the villous and crypt lengths. In addition, it would be of interest to know whether the composition and/or activation status of leukocyte populations in the lamina propria was affected by PcAs infection, although this is perhaps a question for future studies.

**----**

**RESPONSE**:

 (Comment 1) We originally graphed villous height to crypt depth ratio, as this is a common histomorphic measurement used on neighbouring villi and crypts. As the reviewer rightly points out, this measurement cannot differentiate between villous atrophy and crypt hyperplasia. In the new Fig 4, we have now graphed solely villous height and crypt depth. While there is a trend for both villous atrophy and crypt hyperplasia, the data is not significant. In our analysis, differences are only observed when neighbouring villi and crypts are compared against one another. This calculation may be more sensitive, as the Vh:Cd ratio takes into account the proliferation of intestinal progenitor/stem cells within the crypt against the blunting of the neighbouring villous rather than 10/30 randomised single measurements. Pathological changes may not be uniform across the intestinal surface and thus larger sampling may be warranted in future studies. Regardless, the data here suggests that neither villous atrophy nor crypt hyperplasia alone is a dominant phenotype. In all, we would conclude that changes in intestinal pathology during PcAS are mild.

*Updated text for ‘Comment 1’:*

 Results (paragraph 5):

 “However, neither villous height nor crypt depth alone was significantly different in the small intestine.”

 (Comment 2) We agree that understanding the cellular infiltrate would be important. We are actively exploring this avenue in order to fully characterise the source of faecal lactoferrin, which is presumably via an influx of intestinal neutrophils. These points have been expanded on in a new paragraph in the discussion.

*Updated text for ‘Comment 2’:*

 Discussion (paragraph 6):

 “This study reveals novel observational changes in the intestine during a mild, avirulent murine malaria parasite infection. Future work is needed to unpick the mechanism by which intestinal inflammation is induced, and how this relates to intestinal permeability. This would include phenotypical analysis of the cellular source of the inflammatory mediators observed (e.g. IFN-γ, TNF-α, lactoferrin), as well as their location within the tissue and their contribution to intestinal permeability.”

**----**

 Figure 5 presents data showing that PcAS infection also led to increased intestinal permeability, as evidenced by increased levels of plasma FITC-dextran, as well as elevated lactoferrin in the colon contents. Figure 6 extends these analyses by showing that these increases correlated with the levels of parasitaemia, suggesting that they are a consequence of the systemic infection. In this respect, it would be of interest to determine whether plasma IFNg and TNF levels also correlated positively with the measures of intestinal permeability.

**----**

**RESPONSE**:

 We agree, and appreciate that it would be useful to show all parameters in a single figure for related animals. Therefore, we now show circulating IFNγ (measured by ELISA) in Figure 5 and, in Figure 6B, we have plotted this plasma IFNγ against permeability (FITC-Dextran). The data shows that a positive correlation is found only at 9 d.p.i., when IFNγ has diminished. Nevertheless, it is important to recognise that the link between IFNγ and intestinal permeability may not be linear and as the reviewer suggests, it may be important to measure additional inflammatory mediators, such as TNF. We have included additional comments in the discussion (paragraph 6, pasted above), highlighting that a more robust characterisation of proteins pertinent to intestinal permeability, rather than indicative of a generalised inflammatory response such as IFNγ, would be of great interest. We thank the reviewer for his comment and look forward to future research on this topic.

**----**

 Overall, the data are clear, well controlled and very nicely presented. The conclusions made by the investigators are balanced and reasonable and the discussion places them into the wider context and identifies areas for further study.

 The key strengths of the study are that it represents a good model of milder malaria infection and presents clear data and detailed kinetic analyses. The model offers scope for further development to better understand the consequences of altered intestinal permeability on the ongoing malaria infection, as well as predisposition to enteric pathogens.

 The limitations are that the data are largely descriptive and offer limited mechanistic insight. For example, which cytokines are driving the increased intestinal permeability and what are the key cellular sources of these inflammatory mediators? Are they all systemic or are some local to the intestine? The model provides a solid basis for interrogating these and other questions.

**----**

**RESPONSE**:

 Thank you for this very constructive and thoughtful review. Whilst this data does investigate malaria-related inflammation in a new model, we agree that more mechanistic work is now needed to identify the drivers of increased permeability and what, if any, contribution this has on promoting dissemination of secondary enteropathogens.

**----**
